# Sensing and detection performance of the novel, small-diameter OmniaSecure defibrillation lead: in-depth analysis from the LEADR trial

**DOI:** 10.1093/europace/euaf062

**Published:** 2025-03-25

**Authors:** Prashanthan Sanders, Pamela K Mason, Bert Hansky, Paolo De Filippo, Maully J Shah, Darius P Sholevar, John S Zakaib, Francois Philippon, Bernice Tsang, Rajeev K Pathak, Travis D Richardson, Meir Friedman, Robert D Schaller, Ignasi Anguera, Attila Mihalcz, Babak Bozorgnia, Amy E Thompson, Katherin Arias, Baerbel Maus, Chad Bounds, George H Crossley

**Affiliations:** Centre for Heart Rhythm Disorders, University of Adelaide and Royal Adelaide Hospital, Adelaide, South Australia, Australia; Electrophysiology Laboratory, University of Virginia, Charlottesville, VA, USA; Klink für Kardiologie und Internistische Intensivmedizin, Städtische Kliniken, Bielefeld, Germany; Cardiac Electrophysiology and Pacing, ASST Papa Giovanni XXIII, Bergamo, Italy; Cardiac Center, The Children’s Hospital, Philadelphia, PA, USA; Cardiology Group, Virtua Health, Cherry Hill, NJ, USA; Heart Rhythm Center, Minneapolis Heart Institute Foundation, Minneapolis, MN, USA; Institut Universitaire de Cardiologie et de Pneumologie de Québec, Laval University, Québec City, Québec, Canada; Cardiac Program, Southlake Regional Health Centre, Newmarket, Ontario, Canada; Cardiac Electrophysiology & Pacing, Canberra Heart Rhythm Centre and Australian National University, Garran, ACT, Australia; Divsion of Cardiovascular Medicine, Vanderbilt University Medical Center, Nashville, TN, USA; Heart & Vascular Institute Wellness Center, Hartford Hospital, Hartford, CT, USA; Cardiac Arrhythmia Program, Hospital of the University of Pennsylvania, Philadelphia, PA, USA; Unidad de Arritmias y Electrofisiologia, Hospital Universitari Bellvitge, Barcelona, Spain; Klinische Abteilung für Innere Medizin 1, Universitätsklinikum Krems, Krems, Austria; Cardiology Department, Lehigh Valley Hospital—Cedar Crest, Allentown, PA, USA; Cardiac Rhythm Management, Medtronic, Inc., Minneapolis, MN, USA; Cardiac Rhythm Management, Medtronic, Inc., Minneapolis, MN, USA; Cardiac Rhythn Management, Medtronic Bakken Research Center, Maastricht, The Netherlands; Cardiac Rhythm Management, Medtronic, Inc., Minneapolis, MN, USA; Divsion of Cardiovascular Medicine, Vanderbilt University Medical Center, Nashville, TN, USA

**Keywords:** Implantable cardioverter-defibrillator, Inappropriate shocks, P-wave oversensing, Anti-tachycardia pacing, Lumenless leads

## Abstract

**Aims:**

The Lead EvaluAtion for Defibrillation and Reliability (LEADR) trial evaluated the small-diameter (4.7 Fr), integrated bipolar OmniaSecure defibrillation lead. As previously reported, the trial exceeded primary safety and efficacy objective thresholds, demonstrating favourable performance and zero fractures through ∼12 months follow-up, with patients in ongoing follow-up. Longer-term follow-up of the LEADR trial with emphasis on the sensing and detection capabilities of the OmniaSecure lead is reported here.

**Methods and results:**

Patients with indications for *de novo* implantable cardioverter-defibrillators/cardiac resynchronisation therapy defibrillators were implanted with the OmniaSecure lead in standard right ventricle (RV) locations and followed at pre-specified intervals along with CareLink™ remote monitoring transmissions, where available. Throughout follow-up, the lead was evaluated for safety, efficacy, and reliability along with sensing and detection performance. There were 643/657 (97.9%) patients successfully implanted with the OmniaSecure lead with mean follow-up of 18.2 ± 5.5 months. There was a 96.9% freedom from major study lead-related complications at 24 months. Inappropriate shock rate was 2.7 and 3.8% at 12 and 24 months, respectively. At 24 months, 17.6% of patients received appropriate therapies (shock and/or ATP) with a 76.5% ATP efficacy. There have been zero fractures during follow-up along with chronically stable pacing capture threshold, pacing impedance, and R-wave amplitudes. There were four patients with an adverse event related to PWOS (0.6%), none of which was associated with inappropriate shock. There were four patients with an adverse event related to TWOS (0.6%), of which three patients were associated with inappropriate shock (0.5%). Oversensing was resolved predominantly by programming the RV sensitivity to less sensitive settings. During VF induction at implant, 97.6% (120/123) of patients showed appropriate VF episode detection at the least sensitive setting of 1.2 mV, with the remaining having detection at more sensitive settings. In follow-up, 670 VT/VF episodes were appropriately detected and treated in 94 patients with a variety of RV sensitivities and no reports of under-detected episodes. Moreover, a virtual sensitivity analysis also showed no under-detection across different RV sensitivity programming.

**Conclusion:**

Chronic sensing performance of the OmniaSecure defibrillation lead demonstrated R-wave stability with a low rate of P-wave and T-wave oversensing, resolved predominantly by adjusting RV sensitivity. Further, VT/VF detection was successful and was not impacted when programmed to less sensitive settings. The OmniaSecure lead shows robust sensing and detection performance and programmability in ongoing follow-up.

## Graphical Abstract

**Graphical Abstract:**
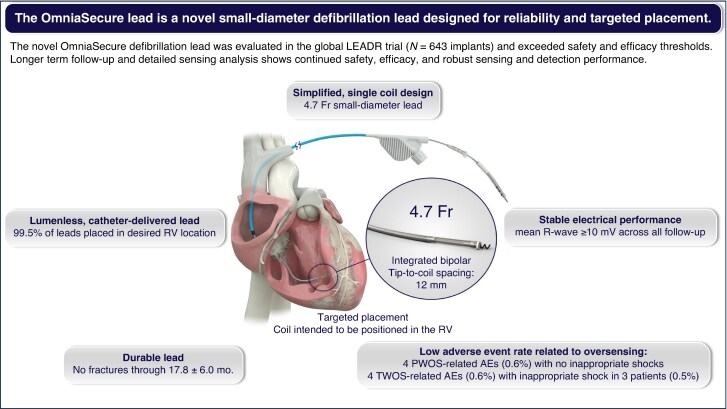

**What's new?**

- Safety and efficacy of the 4.7 Fr ICD lead—the OmniaSecure defibrillation lead.
- No fractures through an average of 17.8 ± 6 months of follow-up in the LEADR trial.
- Stable electrical performance.
- Low incidence of adverse events related to oversensing.

## Introduction

Implantable cardioverter-defibrillators (ICDs) are devices that deliver therapy to convert life-threatening arrhythmias, which are composed of two elements, the pulse generator and the leads. Patients with a high risk of sudden cardiac death may receive an ICD as primary prevention without a history of life-threatening arrhythmias or as secondary prevention for patients with a history of life-threatening arrhythmias.^1^ Despite years of technological advances, the lead remains the ‘Achilles heel’ of the ICD systems due to the nature of the implant conditions and the potential for lead failure that can result in adverse complications for the patient.^2,3^

The OmniaSecure lead, designed for reliability,^4^ is a small-diameter (4.7 Fr), lumenless, integrated bipolar defibrillation lead based on the SelectSecure™ SureScan™ MRI Model 3830 pacing lead that has shown reliable long-term performance.^5–7^ The Lead EvaluAtion for Defibrillation and Reliability (LEADR) trial evaluated the safety and efficacy of the OmniaSecure defibrillation lead. The trial exceeded primary safety and efficacy objective thresholds, demonstrating favourable performance and zero fractures through ∼12 months.^8^ The LEADR trial patients remain in ongoing follow-up. While there are known advantages of integrated bipolar leads, these leads can be associated with oversensing. The purpose of this paper is to report additional follow-up of the LEADR trial with an emphasis on the sensing and detection capabilities of the OmniaSecure lead.

## Methods

### Study design, population, and objectives

The LEADR trial is a prospective, multicenter, single-arm, adaptive, pivotal clinical trial (NCT04863664) with 47 participating study sites worldwide. The trial was approved per local regulatory requirements and ethics committees at each site. After providing written informed consent and in accordance with the Declaration of Helsinki as well as local ethics committees, patients were enrolled if they met ACC/AHA/ESC guideline-directed indications for *de novo* implantation of a primary or secondary prevention ICD or cardiac resynchronisation therapy defibrillator (CRT-D). The primary efficacy objective evaluated defibrillation efficacy at implantation in a subgroup of at least the first 95 consecutive patients completing the testing protocol. Defibrillation efficacy was tested by VF induction, where sensing was evaluated through the ICD/CRT-D for implant with the RV sense value programmed to 1.2 millivolts (mV) per protocol, with allowance to reduce sensitivity value if under-sensing was observed. The primary safety objective evaluated freedom from major complications related to the study lead at 6 months. During continued follow-up, ongoing safety, ambulatory efficacy, and electrical performance were also evaluated.

### Lead and procedure description

**Figure 1:**
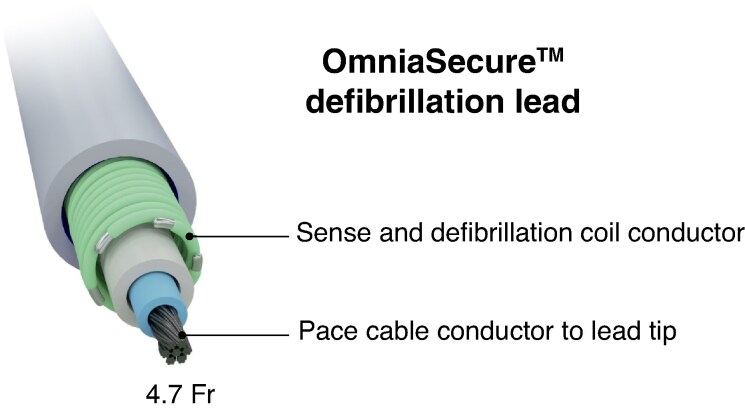
Cross-sectional view of the OmniaSecure defibrillation lead, adapted from Crossley et al.^8^

The OmniaSecure™ SureScan™ MRI defibrillation lead (*Figure 1*) is a 4.7 Fr, integrated bipolar (tip-to-coil polarity), single-coil, lumenless, catheter-delivered, active-fixation transvenous lead with a right ventricular (RV) defibrillation coil electrode (6.1 cm length, 371 mm^2^ surface area, 12 mm tip-to-coil spacing).

Patients were implanted with the lead connected by DF4 connector to a commercially available pulse generator (Medtronic SureScan^TM^ ICD or CRT-D). Lead implantation was allowed only in standard RV positions and not in the left bundle branch locations, per protocol. Care was taken to ensure that the entire defibrillation coil was in the right ventricle after lead tip fixation. Electrical testing included assessment of R-wave sensing, pacing impedance, and pacing capture threshold. Tachyarrhythmia detection and therapies as well as pacing settings were programmed per the physician’s discretion. After implantation, follow-up assessments were conducted at 3 months, 6 months, and will be conducted every 6 months thereafter until study closure. Data were collected at clinical follow-up visits and via CareLink™ remote monitoring transmissions, where available, throughout the study. Throughout follow-up, sensing performance was evaluated by the physician via standard in-office monitoring and via CareLink remote monitoring transmissions (if applicable, i.e. patient on CareLink network), including pacing capture threshold testing and review of real-time EGM, spontaneous episodes, CareAlerts, and other device-recorded data such as sensing integrity counters and pacing percentages. The presence of an integrated bipolar lead continues to underscore the importance of evaluating oversensing in the device function, which includes the auto-adjusting sensitivity feature. Therefore, physicians were trained to carefully monitor and report oversensing, independent of whether it was associated with an adverse event or whether any clinical action was taken.

### Virtual sensitivity analysis

A *post hoc* method was developed to evaluate the safety of the programmable sensitivity settings for detection of VF.^9^ Virtual sensitivity analysis is a method of evaluating ICD device sensing and detection performance of previously recorded spontaneous ventricular arrhythmias. The virtual analysis utilizes data from recorded patient episodes and assesses them at various sensitivity values in the device to determine the potential for under-detection or delays to detection at non-nominal programmable settings.

EGM data were collected via ICD/CRT-D recordings throughout follow-up of the LEADR trial. These spontaneous episodes were adjudicated by an independent Episode Review Committee consisting of physicians to classify the recorded episodes for the true rhythm of each event. From the adjudicated results, episodes classified as polymorphic VF were selected for further analysis, as these types of episodes require high-voltage shock therapy to defibrillate.

Data from these episodes were recorded to the defibrillator, which captures the unfiltered EGM to be used as input for the virtual sensitivity analysis. Episodes with a minimum duration of 30 sensed R-R intervals, exceeding the minimum rate for VF detection by the ICD, were used for virtual analysis. These episodes were processed and input to the virtual simulation system (see Supplementary material online, *Figure S1*), where the ICD component was programmed to nominal VF detection parameters [VF detection zone: Number of Intervals for Detection (NID) at 30/40 with R-R interval ≤ 320 ms]. The detected episodes were recorded from each of the input episodes at each of the following RV sensitivity values: 0.3, 0.45, 0.6, 0.9, and 1.2 mV. The time to detect VF was measured for each tested sensitivity setting for all input episodes used in the simulation. The simulated detection time at 0.3 mV was compared with that of the other sensitivity values. From previous publications, a delay in the detection time was considered clinically significant if >2.5 s.^9^ VF episode under-detection was determined when the device did not reach the 30/40 NID within the input data duration.

### Statistical analysis

Descriptive statistics are presented using mean ± standard deviation for continuous variables, while categorical variables are presented as percentages.

Safety was assessed through freedom from RV-lead related major complications via Kaplan-Meier method, efficacy was assessed as ambulatory therapy efficacy (shock and ATP) during all available device follow-up data using simple proportions, and reliability was assessed through lead electrical measurements and fracture-free performance of the OmniaSecure lead. The rates of appropriate and inappropriate therapy were also determined via Kaplan-Meier analysis. In addition, lead electrical performance was characterized using descriptive statistics. Statistical analyses were performed using SAS version 9.4 and R-version R-4.0.3.

## Results

### Overall clinical trial results

**Figure 2:**
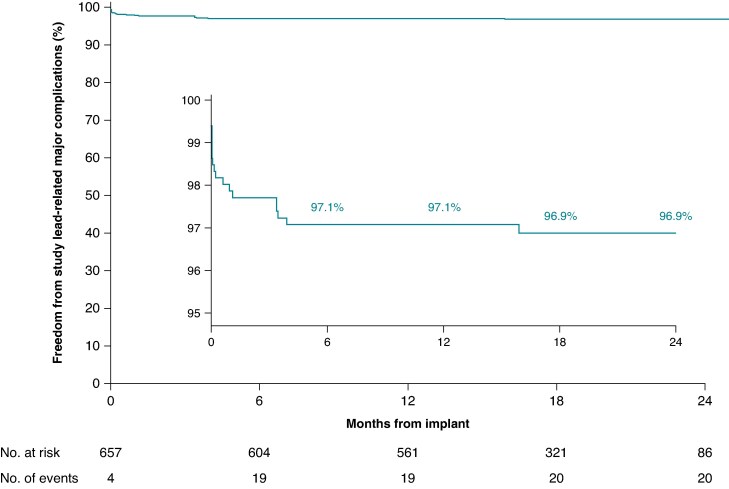
Kaplan-Meier estimated freedom from study lead-related major complications through 2 years post-implant.

**Figure 3:**
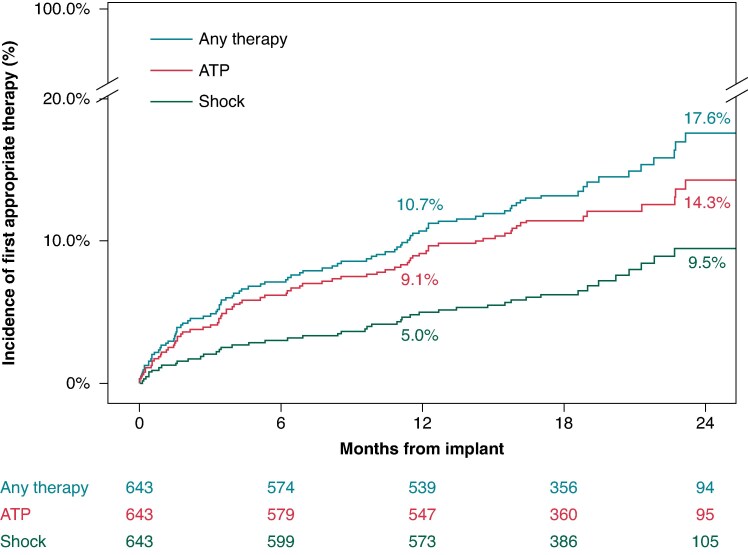
Kaplan-Meier estimated incidence of first appropriate shock, ATP, or any therapy through 2 years. Values in Figure provide rates at 12 and 24 months.

In total, 643/657 (97.9%) patients were successfully implanted with the OmniaSecure defibrillation lead in the standard RV location (26.0% female; 61.9 ± 12.9 years; 35.9% single-chamber ICD, 40.7% dual-chamber ICD, and 23.3% CRT-D), with 99.5% of leads placed in the desired location. The LEADR trial passed the primary efficacy and safety objectives, exceeding the pre-specified thresholds.^8^ The study reported a 97.5% defibrillation efficacy at implant and a 97.1% freedom from study lead-related major complications at 6 and 12 months [95% Confidence interval (CI): 95.4–98.1%]. There was a 96.9% (95% CI: 95.2–98.0%) freedom from study lead-related major complications at 24 months (average follow-up of 17.8 ± 6.0 months) (*Figure 2*). There have been 670 ambulatory VT/VF episodes in 94 patients that received appropriate therapy (shock and/or ATP) through device follow-up of 19.3 ± 5.8 months. Termination of ambulatory events with shock was successful in 94% (124/132 episodes), with the remainder ATP terminated (6), self-terminated (1), or associated with other patient factors (1) (see Supplementary material online, *Appendix*). ATP efficacy was 76.5% (485/634 episodes) in 76 patients. A total of 10.7% (95% CI: 8.5–13.4%) of patients received an appropriate therapy by 12 months and 17.6% (95% CI: 14.2–21.7%) by 24 months (*Figure 3*). The inappropriate shock rate was 2.7% at 12 months (95% CI: 1.7–4.3%) and 3.8% at 24 months (95% CI: 2.4–5.9%), see Supplementary material online, *Appendix* for further details. There were zero fractures of the OmniaSecure lead through 17.8 ± 6.0 month follow-up.

### Electrical performance

**Figure 4:**
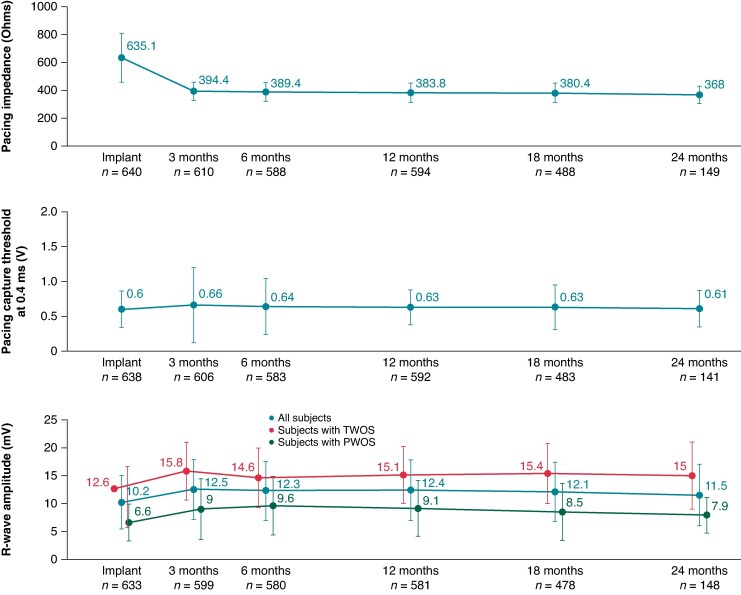
Electrical measurements for pacing impedance, pacing capture threshold, and R-wave amplitude through 24 months. Mean ± standard deviation is presented for each visit. The values in the figure provide the mean while the errors bars represent the standard deviation.

During follow-up, the functional electrical performance of the OmniaSecure defibrillation lead was evaluated (*Figure 4*). The mean pacing impedance was 635.1 ± 175.0 Ohms at implant, 394.4 ± 64.5 Ohms at 3-month follow-up, and remained stable thereafter. The mean pacing capture threshold was 0.60 ± 0.26 V at implant and remained stable throughout follow-up.

The OmniaSecure lead sensing was evaluated by R-wave amplitude monitoring at implant and through follow-up. The recommended implant acceptance criteria for R-wave amplitude was ≥5 mV. The R-wave amplitude at implant was on average 10.2 ± 4.8 mV (25–75th percentile: 6–13 mV) and remained stable throughout follow-up.

### Physiologic oversensing

While sensing was adequate and remained stable, oversensing was occasionally observed in some patients. Physicians were trained to carefully monitor and report oversensing, independent of whether it was associated with an adverse event or if any subsequent action was taken. Overall, there were 74 patients with reports of physiologic oversensing, irrespective of time since implant or clinical implications (PWOS *N* = 19, TWOS *N* = 38, TWOS and PWOS *N* = 1, R-wave double counting *N* = 4, and unspecified *N* = 14; see Supplementary material online, *Appendix*). The vast majority of patients, 87.8% (65/74), did not have an adverse event associated with oversensing. The breakdown by device type is shown in *Table 1*.

**Table 1:** Breakdown of device type for the overall LEADR trial and for those patients with reports of oversensing

	Single chamber ICD	Dual chamber ICD	CRT-D
All LEADR patients (*N* = 643)	35.9% (231)	40.7% (262)	23.3% (150)
PWOS (*N* = 19)^a^	26.3% (5)	57.9% (11)	15.8% (3)
TWOS (*N* = 38)^a^	26.3% (10)	23.7% (9)	50% (19)
PWOS and TWOS (*N* = 1)	N/A	N/A	100% (1)
R-wave double counting (*N* = 4)	75% (3)	N/A	25% (1)
Unspecified (*N* = 14)^a^	50% (7)	43% (6)	7% (1)

^a^There were 74 unique patients with physiologic oversensing, one patient had PWOS in addition to unspecified oversensing and another patient had TWOS in addition to unspecified oversensing.

Nineteen patients had reports of PWOS, of which eight patients had reports at implant and 11 patients at follow-up. Importantly, there were no inappropriate shocks due to PWOS. The average R-wave amplitude for the 19 patients with PWOS was stable through follow-up (*Figure 4*). PWOS could be mitigated via programming adjustments in 17/19 patients (ICD: *N* = 14 and CRT-D: *N* = 3). Of the remaining two, one patient had the lead repositioned at implant, and the other had the lead replaced in chronic follow-up (more information below).

Four (0.6%) of the nineteen patients had a PWOS-associated adverse event. Of the four patients with a PWOS adverse event, one patient had PWOS resolved by programming the RV sensitivity to 1.2 mV with sensing confirmed at this sensitivity value via VF induction in hospital (34 days post-implant), another patient had a system revision to replace the lead with a non-study lead (119 days post-implant), and the remaining two patients showed PWOS resolution by programing adjustments, however underwent an elective replacement of the lead with a non-study lead at physician discretion (at implant and 1 day post-implant). (See Supplementary material online, *Appendix* for follow-up details pertaining to the remaining patients that did not have an associated adverse event).

Among patients with PWOS that did not undergo a lead replacement, the final RV sensitivity at most recent follow-up was 0.3 (2), 0.45 (5), 0.6 (4), 0.9 (3), or 1.2 mV (2). Of the two patients programmed at 1.2 mV RV sensitivity, induction testing at this setting was completed for one (due to PWOS adverse event).

Thirty-eight patients had reports of TWOS, of which six patients had reports at implant and 32 patients at follow-up. Among these, there were only three patients with inappropriate shock due to TWOS, 0.5%, subsequently described. The average R-wave amplitude for the 38 patients with TWOS was stable through follow-up (*Figure 4*). There were 21 patients with TWOS characterized as post-pace TWOS, 16 patients with post-sense TWOS, and one patient with both post-pace and post-sense, see *Table 2* for breakdown by device type. TWOS was resolved via programming adjustments in 34 of 38 patients (ICD: *N* = 16 and CRT-D: *N* = 18). Of the remaining, one resulted in an unsuccessful implant, one was resolved via repositioning at implant, one had no clinical action taken (387 days post-implant), and one patient had the lead replaced (described below).

**Table 2:** Distribution of post-pace and post-sense TWOS by device type

	Single chamber ICD (*N* = 10)	Dual chamber ICD (*N* = 9)	CRT-D (*N* = 19)
PP-TWOS (*N* = 21)	9.5% (2)	19% (4)	71.5% (15)
PS-TWOS (*N* = 16)	50% (8)	25% (4)	25% (4)
PP and PS-TWOS (*N* = 1)	N/A	100% (1)	N/A

Four (0.6%) of the 38 patients had a TWOS-associated adverse event. Of these four patients, one patient had a system revision to replace the lead with a non-study lead due to post-pace TWOS that could not be resolved with programing adjustments (359 days); however, TWOS continued after replacement with a non-study lead, requiring further revision of lead placement (see Supplementary material online, *Appendix*). Three patients had an inappropriate shock due to post-sense TWOS (118, 374, and 630 days) that were mitigated by programming adjustments, after which no further events have been reported. (See Supplementary material online, *Appendix* for follow-up details pertaining to the remaining patients that did not have an associated adverse event).

Among patients with TWOS that did not undergo a lead replacement, the final RV sensitivity value at most recent follow-up was 0.3 (1), 0.45 (22), 0.6 (12), and 1.2 mV (1). In the patient programmed to 1.2 mV, induction testing was not conducted; however, there was a spontaneous episode successfully detected and treated while programmed at 1.2 mV.

#### Tachyarrhythmia detection performance

During induced VF as part of defibrillation testing at implant, 97.6% (120/123) of patients showed appropriate detection at the least sensitive setting (1.2 mV) with the remaining three successful at more sensitive settings. Among these three, detection was confirmed at 0.9 mV for one patient while the other two patients were adjusted to 0.3 mV without further testing at other sensitivity values, per physician discretion (see Supplementary material online, *Appendix* for further information). There were 670 ambulatory VT/VF episodes appropriately treated in 94 patients, all of which were successfully detected across a variety of programmed sensitivities (*Table 3*) with no reports of under-detection of spontaneous VT/VF episodes.

**Table 3:** Proportion of successfully treated ambulatory events by programmed RV sensitivity

Available Programing (mV)	Programming at time of successfully treated ambulatory event (*N* = 670 episodes in 94 patients)
0.3 [nom]	69.3%
0.45	21.9%
0.6	8.7%
0.9	0.0%
1.2	0.2%

#### Virtual sensitivity analysis results

There were 33 episodes from 20 patients adjudicated to have true polymorphic VT/VF that could be processed and input to the simulation test environment for the virtual sensitivity analysis. The simulated time to VF detection increased by ≤0.7 s on average from the nominal (0.3 mV) to the highest sensitivity value (1.2 mV) (*Table 4*). The difference in simulated time to detection between the nominal and the next sensitivity level (0.45 mV) was on average 0.11 s and at the maximum 0.58 s. There were no cases of complete under-detection at any RV sensitivity level.

**Table 4:** Virtual sensitivity analysis results

RV sensitivity	Proportion of clinical programming level (*N* = 33 episodes)	Simulated time to VF detection (s) [mean ± SD]	Range of simulated time to VF detection (s) [Min–Max]	Difference in simulated detection of clinical VF time from simulated detection of clinical VF time at 0.3 mV (s) [mean ± SD]	Proportion of episodes with difference in simulated detection of clinical VF time at 0.3 mV > 2.5s
0.3	28/33	7.06 ± 1.66	4.97–11.60	N/A	N/A
0.45	5/33	7.17 ± 1.66	4.96–11.59	0.11 ± 0.19	0/33
0.6	0/33	7.22 ± 1.58	5.07–10.78	0.16 ± 0.34	0/33
0.9	0/33	7.44 ± 1.76	5.07–11.08	0.38 ± 0.55	0/33
1.2	0/33	7.76 ± 2.27	5.06–13.25	0.70 ± 1.70	2/33

## Discussion

In ICD systems, the transvenous lead has remained the ‘Achilles heel’ of the system due to the frequency of lead-related complications.^2,3^ The novel, lumenless, integrated bipolar, small-diameter OmniaSecure defibrillation lead may address this need due to the potential of small-diameter leads having reduced lead-related complications.^10–12^ Moreover, the OmniaSecure lead is based on the SelectSecure 3830 pacing lead that has demonstrated reliable long-term performance.^5–7^ The LEADR trial was conducted to evaluate the safety, efficacy, and reliability of the OmniaSecure lead implanted in the standard RV location.^4^ The primary efficacy and safety objectives were met, and results exceeded the pre-specified performance thresholds.^8^ The LEADR trial continues to demonstrate ongoing safety, efficacy, and reliability of the novel OmniaSecure lead through longer-term follow-up. Chronic sensing performance of the OmniaSecure defibrillation lead demonstrated R-wave stability with a low rate of P-wave and T-wave oversensing, predominantly resolved by reprogramming RV sensitivity. Furthermore, VT/VF detection was successful and was not impacted when reprogrammed to less sensitive settings. The OmniaSecure lead shows robust sensing and detection performance and programmability in ongoing follow-up.

The OmniaSecure lead has a simplified lumenless, single-coil design that enables a small diameter of 4.7 Fr. This simplified lumenless design replaces the central stylet lumen with a fracture-resistant flexible cable conductor that improves durability.^12^ In addition, the OmniaSecure lead incorporates a helix design similar to the SelectSecure 3830 pacing lead to preserve capture threshold performance. There have been no fractures of the OmniaSecure lead through available clinical follow-up, and reliability modelling has projected the 10-year fracture-free survival rate of the OmniaSecure lead to be 98.2%.^12^ The LEADR trial demonstrated average R-wave amplitudes of >10 mV; R-wave amplitude along with pacing capture threshold and pacing impedance remained stable throughout follow-up.

The OmniaSecure lead is an integrated bipolar lead.^4,8^ Literature notes many advantages of an integrated bipolar lead design, including the ability to downsize the lead and thereby reduce patient complications,^4^ increased reliability due to a simplified design,^13^ and increased R-wave amplitudes due to wider electrode spacing.^14–16^

However, integrated bipolar leads can be prone to oversensing.^17–21^ Physiologic oversensing, namely PWOS and TWOS, while rare, may result in inappropriate therapy and/or inhibition of pacing. In most cases, oversensing can be mitigated by adjusting the sensitivity of the ventricular lead with considerations for tachyarrhythmia detection.^22^

In the LEADR trial, PWOS was observed, however, the overall rate of PWOS associated with an adverse event was low at 0.6% (4/657), and there were no inappropriate shocks related to PWOS. TWOS was also reported with a low overall rate of TWOS being associated with an adverse event of 0.6% (4/657), including three patients that had inappropriate shock due to TWOS. This TWOS-related rate of patients with inappropriate shock is in line with the PainFree SST study that included predominantly true bipolar ventricular sensing leads as well as the transvenous arm of the PRAETORIAN trial.^23,24^

Programming adjustments are an effective tool for mitigating oversensing. In the LEADR trial, oversensing was resolved via programming, mitigating PWOS in 89.5% (17/19) of patients and TWOS in 89.5% (34/38) of patients where reprogramming was attempted. The RV sensitivity value of 0.45 mV was the most common programming adjustment that resolved the oversensing events. When adjusting RV sensitivity, there should also be consideration for tachyarrhythmia detection. Through follow-up, all of the 670 ambulatory VT/VF episodes appropriately treated were successfully detected across a variety of programmed ventricular sensitivities with no reports of spontaneous ventricular arrythmia under-detection.

Virtual sensitivity simulation is an effective tool to evaluate the sensing and detection performance at various RV sensitivity values for a given episode with recorded EGM data from the study. There was no under-detection in the simulations regardless of RV sensitivity value programming. When sensitivity was changed from 0.3 to 0.45 mV, the most common adjustment observed in the trial, the time to detection minimally increased, by an average of 110 ms, which is a clinically insignificant delay.^9^ Even when the sensitivity was increased to 0.6 mV, these favourable observations were maintained. These data support the option to program to a progressively less sensitive setting to optimize sensing, with minimal impact on VF detection. Therefore, clinicians may consider programming the RV sensitivity to 0.45 mV, which may be an effective means of proactively mitigating potential oversensing. Overall, this detailed analysis of sensing and detection performance demonstrates a low rate of sensing-related adverse events and the flexibility to resolve sensing observations non-invasively through programming.

## Limitations

The LEADR trial remains in ongoing follow-up and not all patients have reached the 24-month follow-up. Therefore, 24-month results may vary slightly as follow-up continues. Average follow-up time was 17.8 ± 6.0 months, including 153 patients with 24-month follow-up and 24 patients with 30-month follow-up. The OmniaSecure lead is a DF4 only configuration, and comparisons to leads with other connector types were not evaluated.

Observed oversensing in this study was as reported by the study investigators for whom all study data from follow-up and CareLink transmissions (if applicable) were made available. However, there may be variation among investigators for the criteria of reportable oversensing.

The virtual sensitivity simulations were restricted to recorded episodes adjudicated as polymorphic VT/VF; this limited the sample size to 33 episodes from 20 patients in the trial. Therefore, these results may not be generalizable to all arrhythmias or to all patients in the study.

Sensing was evaluated with the OmniaSecure lead in combination with a Medtronic ICD/CRT-D; therefore, extrapolating results individually to the lead or the device may not be possible since both were studied as part of a whole system.

## Conclusion

The LEADR trial demonstrates safety, efficacy, and reliability of the novel, small-diameter OmniaSecure lead, with low complication rates, high ambulatory efficacy, and reliable performance. Chronic sensing performance of the OmniaSecure defibrillation lead demonstrated R-wave stability with a low rate of oversensing, predominantly resolved by adjusting the RV sensitivity programming, with no clinically relevant impact. The OmniaSecure lead shows robust sensing and detection performance and programmability.

## Supplementary Material

euaf062_Supplementary_Data

## Data Availability

All data requests will be made through the corresponding author. Data requests will require details of the analysis and usage to be performed and will need to be approved by the study steering committee. If approved and within the confines of the Human Research Ethics approval, data will be made available.
