# Supplementary material for: Sensing and detection performance of the novel, small-diameter OmniaSecure defibrillation lead: in-depth analysis from the LEADR trial
Source: Europace. 2025 Mar 25;27(4):euaf062. doi: 10.1093/europace/euaf062 (PMC12001238; doi:10.1093/europace/euaf062)
Supplement: euaf062_Supplementary_Data [file euaf062_supplementary_data.docx]

**Supplementary Appendix**

Sensing and Detection Performance of the Novel, Small-diameter OmniaSecure Defibrillation Lead: In-depth analysis from the LEADR Trial

**Figures**

Supplementary Figure 1: The Virtual Sensing Simulation System


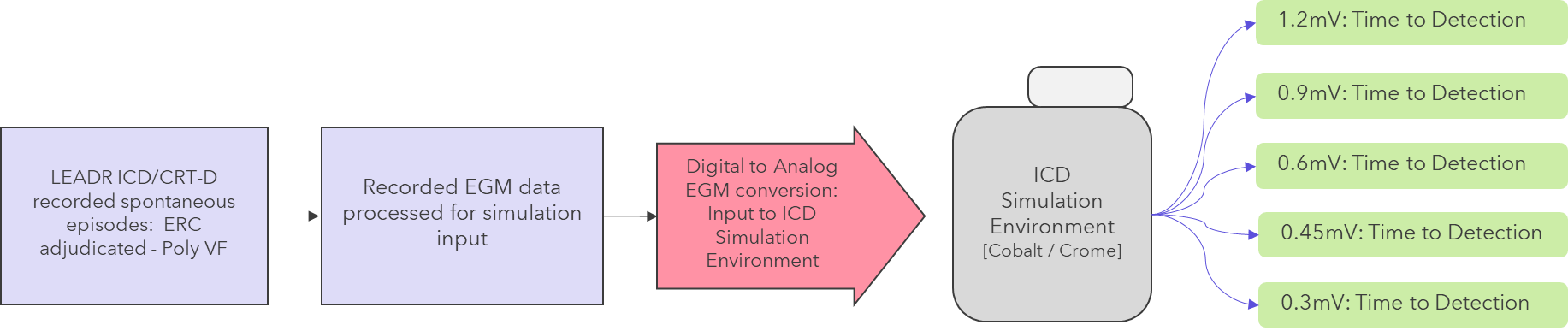


Supplementary Figure 2: Examples of Physiologic Oversensing Observed.


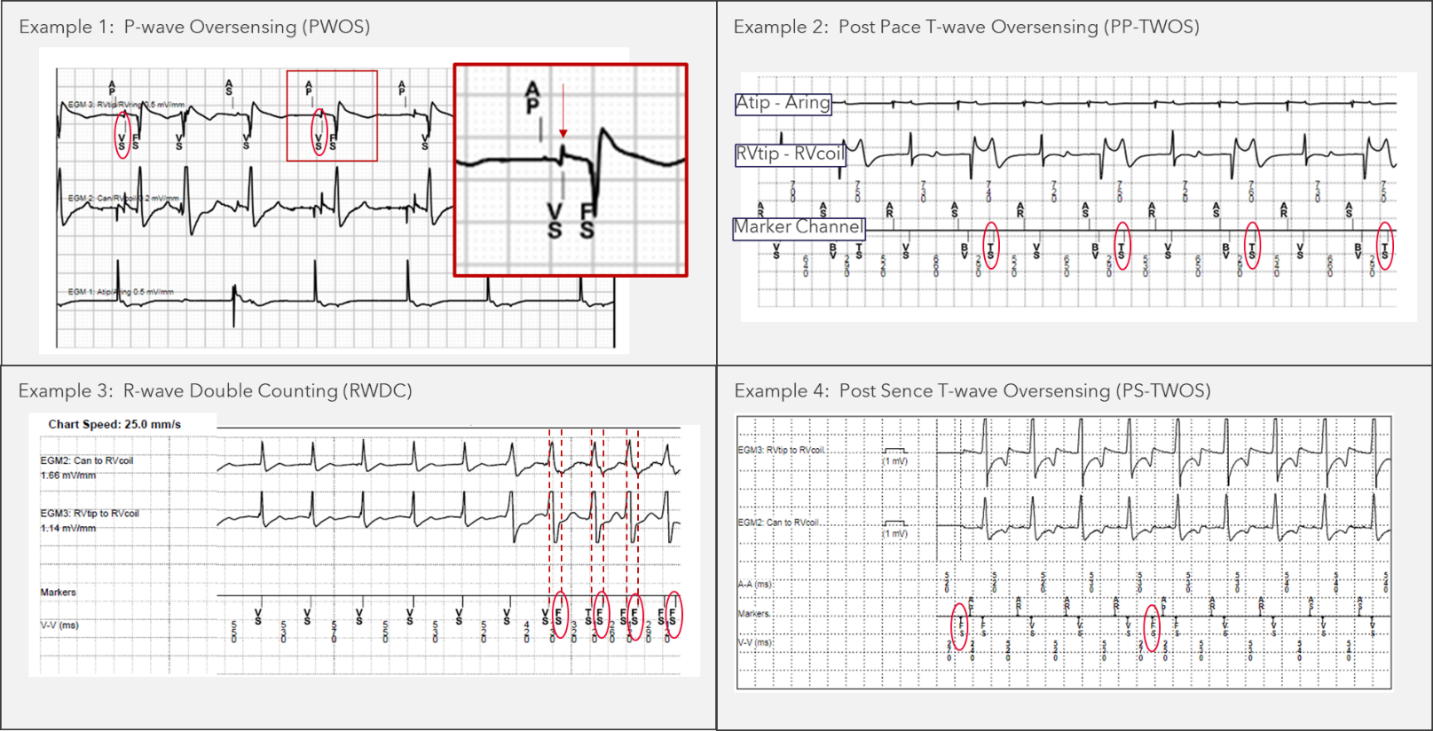


**Results**

*Clinical Trial Results*

Of the 8 episodes not successfully treated with shock, 3 episodes had the rhythm slowed to be ATP terminated,  3 episodes (2 patients) occurred in the context of a storm with the final episode ATP terminated, 1 episode self-terminated and 1 episode of polymorphic VT/VF failed to convert after delivering all available shocks on the day of the patient’s death. This patient had cardiac amyloidosis and endocardial scarring of both ventricles.

Inappropriate therapies were those episodes with a true dominate rhythm adjudicated by the independent physician episode review committee (ERC) as Non-sustained VT, Atrial fibrillation/Atrial flutter, Noise rhythms (P-wave oversensing, T-wave oversensing, and non-physiologic oversensing), Other Supraventricular Tachycardia/Sinus Tachycardia, or Other non-VT/VF.

The rate of patients with inappropriate shock was 2.7% at 12 months (95% Confidence interval: 1.7% - 4.3%) and 3.8% at 24 months (95% Confidence interval: 2.4% - 5.9%). There were 21 patients (7 single chamber and 14 dual/triple chamber) that had 40 episodes with inappropriate shock through average device follow-up of 19.3 +/- 5.8 months. The causes of inappropriate shock were Atrial Fibrillation/Atrial Flutter (26), Other SVT or sinus tachycardia (5), TWOS (4), OmniaSecure lead dislodgement (3), non-cardiac oversensing (1), and Cardiogenic shock death (1).

*P-wave and T-wave Oversensing*

There were 74 patients with reports of oversensing regardless of clinical implications with PWOS N=19, TWOS N=38, TWOS & PWOS N=1, R-wave double counting N=4, and unspecified N=14. See **Supplementary Figure 2** for examples of physiologic oversensing.

Of the 19 patients with reports of PWOS, 15 patients had instances of PWOS noted by physicians but were not associated with an adverse event (7 patients at implant and 8 patients at follow-up). These additional instances could be resolved via lead repositioning at implant to ensure the entire coil was in the ventricle (N=1) or programming adjustments (N=14).

There were no inappropriate shocks due to PWOS. The independent physician episode review committee (ERC) adjudication distinguished between rapid ventricular rate from conducted AF and PWOS of AF on the RV sensing channel.

In total, PWOS could be mitigated via programming adjustments in 17/19 patients. Among these, only 1 patient needed a second programming adjustment to resolve PWOS thereafter; for all others, no further PWOS was reported after initial programming adjustment. There were two patients that underwent other programming mitigations besides RV sensitivity adjustments including changes to the atrial pacing output and RV sense blanking after ventricular pacing.

Of the 38 patients with reports of TWOS, 34 patients had instances of TWOS noted by physicians but not associated with an adverse event (6 patients at implant and 28 patients at follow-up). Of these additional instances, 1 resulted in an unsuccessful implant and 1 had no programming adjustments in response to the observation (387 days postimplant); the others were resolved via repositioning at implant (1) and programming adjustments (31). For the patient that had lead repositioning at implant, there were no further reports of TWOS thereafter and no further programming mitigations.

In total, TWOS could be mitigated via programming adjustments in 34/38 patients, including the three patients with inappropriate shock due to TWOS. In each case, the initial programming adjustment was to increase the RV sensitivity value. Six patients required other programming adjustments including four CRT-D patients who underwent programming mitigations such as changing the RV-LV pace delay and BiV pacing mode.

One patient that underwent a lead replacement due to unresolvable TWOS continued to exhibit TWOS behavior when the replacement non-study lead was positioned in a similar location. Thus, the observed TWOS may not have been attributable to the OmniaSecure lead. The resolution was to place the replacement lead in the RVOT which alleviated TWOS.

In addition to the patients described above, there was 1 patient with both reported PWOS and post-pace TWOS at follow-up, both resolved via adjusting the RV sensitivity to 0.6mV with no further reports of oversensing.

*Other Physiologic Oversensing*

There were 4 patients with reports of R-wave double counting noted by physicians via monitoring that were not associated with an adverse event; all were resolved via programming adjustments with no further reports of oversensing thereafter. Three patients had the RV sensitivity value adjusted to 0.45 mV and one patient had a mitigation by programming the RV sense blanking after ventricular sensing to 140ms from the nominal 120ms value.

There were 14 patients with reports of oversensing that were classified as unspecified, as there were device monitoring data available that was consistent with oversensing but without EGM data available to confirm the specific type of oversensing. Of these, one was associated with an adverse event resulting in replacement with a non-study lead. The remaining 13 patients had instances of unspecified physiologic oversensing noted by physicians that were not associated with an adverse event. Among these, 1 had no programming adjustments in response to the observation while the others were resolved via repositioning at implant (1) and programming adjustment (11). One patient that had the lead repositioned at implant had no further reports of oversensing thereafter and no further programming adjustments typically associated with oversensing. Unspecified physiologic oversensing was resolved via programming mitigations in a total of 11 patients (5 single chamber ICD and 6 dual chamber ICD). One patient needed further programming adjustments. Of the 11, 9 patients had adjustments to the RV sensitivity value and 2 patients had adjustments to RV sense blanking after ventricular sensing. Among patients with unspecified physiologic oversensing that did not undergo a lead replacement, the final RV sensitivity at most recent follow-up was 0.3 (4), 0.45 (5), and 0.6 (4) mV.

*Other Oversensing*

There was one additional patient with non-physiologic oversensing that was seen while measuring lead impedance in office (CRT-D device without an atrial lead). This can happen very infrequently and does not result in any clinical impact on the device; therefore, no action was taken.

There was also one patient with oversensing of electromagnetic interference from the patient manipulating an electrical device in a swimming pool. This electromagnetic signal was detected by the device as a fast rhythm which resulted in an inappropriate shock that was classified as an adverse event.

*Tachyarrhythmia Detection performance*

Of the three patients that showed appropriate detection at lower sensitivities, 0.3 mV (2) and 0.9 mV (1) during induced VF as part of defibrillation testing at implant, the final RV sensitivity was programmed to 0.3 mV at implant. During follow-up, one patient had TWOS noted by the physician but not associated with an adverse event which was resolved by adjusting the RV sensitivity to 0.45 mV.
